# The Institutional Worker

**Published:** 1921-03-05

**Authors:** 


### THE INSTITUTIONAL WORKER.

*Being a Special Supplement to "The Hospital."*

### INSTITUTION FACTS AND FIGURES.

### The Work of an Inquiry Officer.

By ODD NO.

In a State- or municipal-supported institution the life of the Inquiry Officer may be an easy one; but in the large number of struggling voluntary hospitals he is engaged in the Herculean task of penetrating a veritable stronghold of apathy—a voice crying in the wilderness of ignorance of the real facts of hospital finances. His primary duty is to ascertain the social status of all patients seeking admission to his institution to keep a register of in- and out-patients, and to record information regarding the length of time in hospital, date of discharge, etc. He is also called upon to bring home to each person admitted the obligations which are incurred in the way of maintenance, housing, and nursing. How widespread, alas, is the idea that hospitals receive more than sufficient support from some mysterious source, and are in such a fortunate position financially that no efforts are required on the part of patients themselves to assist in the costly work of carrying on. The recent enviable experience of certain London hospitals in receiving anonymous donations of £500 from a mysterious and unknown stranger seems to lend colour to such a desirable circumstance.

The Inquiry Officer, then, is a man burning with a sense of the injustice wrought to a good cause—chiefly through ignorance of the real facts. His is the duty to put the case of the hospital and its needs before all and sundry—not when they are acutely ill, but rather when returning health brings in its train a sense of deep gratitude. More than half of the financial difficulties of hospitals to-day arise from the fact that these institutions do not cry loud enough in their appeals for funds, they simply do not let the public know what valuable work is being done within their walls. The Inquiry Officer, if he fulfils the requirements of his office, will supply a long-felt want in this direction. How many people outside the hospital circles know of the really vast amount of work done for the public good in the way of preventive medicine, research work, and in many other branches of medical science? whilst the larger hospitals are responsible for the training and education of the men and women who will form the personnel of the medical and nursing professions of to-morrow. With all these facts at his disposal the Inquiry Officer has the material for a very strong appeal.

There are many excuses for evasion of payment for treatment—even from those who are in a position to contribute, at any rate, a portion of the cost incurred; but a keen and imaginative officer should be able to detect any genuine cases of poverty.

These being the main ideals which this officer must bear in mind, might he not be of really much greater service to the institution he serves? What fields are open to him? Each patient should be interviewed and the possibility of a subscription or donation ascertained. If the patient is an employee the name of the employer should be recorded, and an accurate record kept of all persons treated from each individual firm. If these firms are not among the subscribers to the hospital, or if they are sending more patients for treatment than are warranted by the amount of their contribution, the Inquiry Officer should send a letter to them plainly stating to what extent they are making use of other people's subscriptions. This method is responsible for a large percentage of the new subscriptions obtained each year in many hospitals—especially those in industrial districts. The patients themselves seldom turn a deaf ear to the pleading of the Inquiry Officer, and a large sum may be obtained each year from their contributions.

A keen man with a wide imagination will be quick to see and grasp every opportunity for raising funds for the hospital in his association with a very varied list of patients. A professional footballer may be admitted for the treatment of a fracture or other injury sustained in the course of play. While the man himself may not be disposed to make any contribution towards the cost of his treatment, it is within the knowledge of the writer that one large football club—solely through the efforts of the Inquiry Officer—has stepped into the breach with a handsome donation.

The hospital collectors and canvassers will find in this officer a rich mine of valuable information concerning possible subscriptions which can always be followed up with advantage. The collector is usually able to go a step farther than the Inquiry Officer, in being able to track the "fox to his lair," by making personal appeals to employers and others beyond the reach of the Inquiry Officer himself.

Another very valuable source of information this officer might do well to explore is the classification of patients as coming from works, streets (accidents, etc.), or private homes. In connection with this subject, it will be found interesting to compute the total number of patients treated each year for the municipality, the Watch or Police Committee, and for the accident and life insurance companies or other similar bodies. That there is money to be obtained from these sources is exemplified by the fact that recently the Corporation of Liverpool has taken power to contribute £5,000 per annum to their infirmary, and at Salford the produce of a rate of 1d. in the £ is to be handed to the local voluntary hospitals.

The following typical cases illustrate the possibilities of an efficient registration system: A motor accident occurs, and the injured are taken to the nearest hospital for treatment. Although they are almost certainly covered by insurance, no payment is made to the hospital for its services in healing the injured and thus reducing the amount to be paid out by the insurance companies themselves. The workman claims compensation under the Workmen's Compensation Act, when injured in a factory accident; but the hospital is forgotten. Can it claim nothing? Yet another instance: A man attempts to commit suicide, is brought to the hospital and slowly nursed back to health, fed, housed, and watched. When the question of payment is raised the Watch Committee too often say they have no power to make any grant towards the cost of treatment.

Let the Inquiry Officer, then, direct attention to the often-forgotten fact that if it were not for the service rendered to the public by the voluntary hospitals there would in all probability be an increase in the amounts of funds to be paid out in compensation for sickness and accident cases, with a consequent increase in all premiums charged. The insurance companies might well be approached in this connection in view of the very great relief rendered to them by the hospitals throughout the country in the treatment of insured persons.

The general public are ready and willing to subscribe to hospitals for sick and poor persons, but many are withholding support to-day on account of the large number of patients being treated who can afford to pay or be paid for. The keen hospital administrator should see to it that no possible source of income remains untapped, and the Inquiry Officer may in this direction prove himself of inestimable value to the hospital he serves.

### Question for March.

### WARD INVENTORIES.

**What is the simplest method of making an inventory of linen, crockery, furniture, etc., of wards and Nurses' Home? Give directions and examples, and state whether lists should be printed and hung in the wards and home.**

**(A minimum payment of 10s. 6d. will be made for each published answer.)**

RULES.

The following rules must be observed :—

1. Contributions must be written on one side of the paper. Conciseness and terseness are desirable features. The MS. must bear the name and address of the sender and be accompanied by coupon to be cut from the back cover (inside page) of the current issue of The Hospital. A pseudonym must be chosen if the name is not to be published.

2. Contributions must be addressed to the Editor of The Hospital Institutional Worker Supplement, 28 & 29 Southampton Street, Strand, London, W.C. 2, should reach him before the end of the current month, and be marked in the left-hand corner "Facts and Figures."

### ENQUIRIES AND ANSWERS.

*THE SICK AND IN NEED.*

### Private Asylums.

We give you the names of a few private asylums in London for which you ask : Camberwell House, Camberwell, S.E. ; Chiswick House, Chiswick ; Northumberland House, Green Lanes, Finsbury Park, N. ; and Hendon Grove, Hendon, N.W. Write to the Superintendent in each case.—Ida.

### Help for Working Women.

The Secretary of the Women's Holiday Fund would possibly help the woman who is so badly in need of a holiday. The object of the Holiday Fund is to send for a short holiday to the seaside or country hard-working and deserving women who cannot afford the expense a holiday entails. Applicants are, however, required to pay a small sum towards their expenses, according to their means. The address of the Society is 76 Denison House, Vauxhall Bridge Road, S.W. 1.—Mrs. G.

### Hospital for Incurables, Melbourne.

The only hospital for incurables we know of near to Melbourne is the Austin Hospital for Incurables, Heidelberg, near Melbourne. If you write the Secretary-Superintendent he will no doubt give you particulars of admission. His address is Wm. J. E. Turner, 360 Collins Street, Melbourne. —Anxious Parent.

*EMPLOYMENT AND TRAINING.*

### Training as Male Nurse.

Probably your best course would be to apply to the National Hospital for Paralysed and Epileptic, Queen Square, Bloomsbury, W.C., where men between the ages of twenty-one and thirty years are, if suitable, trained for a period of two years. At St. Bartholomew's Hospital a few men are trained for the service of the hospital. Full training in all branches of nursing can be obtained in the military hospitals. This necessitates enlistment as a soldier of the Royal Army Medical Corps. As a rule the term is for a period of twelve years, three of which are usually with the Colours and nine in the Army Reserve. Men desirous of joining the Royal Army Medical Corps with a view to becoming members of the nursing section of the corps should present themselves at any military barracks in the United Kingdom. We do not give postal replies.—A. E. D.

*MISCELLANEOUS.*

### The People's League of Health.

The series of thirteen educational health lectures by Sir Sims Woodhead, arranged under the auspices of the People's League of Health, commenced on February 9. You should have made application sooner, as only a limited number of students can be enrolled for each series. The fee for the series is 10s. Write to the Hon. Organiser, Sims Woodhead Lecture Series, People's League of Health, 7 Hanover Square, W. 1.—"Health."

### Personalia.

Benefactions for Nursing.

Two Bolton families—those of Mr. J. P. Haslam and the late Sir John Scott—retiring from participation in the affairs of the John Haslam cotton factories by reason of the sale of their interests to a new organisation, have marked the occasion by munificent gifts to the amount of £50,000. Of this the sum of £30,000 has been handed over to the Committee of the Bolton Infirmary to build the new Park Road Nursing Home. Mr. J. P. Haslam recently gave his fine house and grounds—Ravenswood—for the purpose of a maternity home.

A Well-managed Nursing Association.

Miss Wilmer holds the honour of having conducted the affairs of the Birkenhead and District Nursing Society as Honorary Treasurer for a period of twenty-four years, during which she has always presented a balance on the right side. She now retires from office, and was presented with an address and a silver tea-service as a mark of appreciation from her colleagues. Nearly 1,000 patients were attended to during the year, and both fees from patients and donations reached a record figure.

A Committee of Patients.

Manor House Hospital at Golder's Green possesses, writes a correspondent, a committee composed of a number of patients, who make suggestions for alterations and improvements. The institution, which has been established recently, is being run on a friendly society basis. It is stated there are some 100,000 subscribing members who pay one penny per week, a sum which entitles them to free treatment when occasion arises. It is gratifying to learn that the undertaking so far is "paying its way."

Doctor as Hospital President.

Dr. H. Keighley, who has been a member of the honorary medical staff for over twenty-five years, has been elected President of the Batley and District Hospital. When accepting the honour Dr. Keighley said that, while many hospitals would lose more than they would gain from any State aid, if rate or State aid were adopted he thought that the fairest method would be to grant it in proportion to the amount of the voluntary subscriptions.

£10,000 for Nottingham Hospital.

Sir John Robinson, of Worksop Manor, an old friend of the institution, has given to the Nottingham General Hospital the sum of £10,000, in memory of his son, the late Mr. John Sandford Robinson. Sir John, who is known for his racing stud at Worksop and his interest in agriculture, married in 1867 the great-granddaughter of that Dr. Davidson who was the first physician appointed to the Nottingham General Hospital. This generous gift is to be recorded on a tablet in the institution.

Central Midwives Board for Scotland.

At the first meeting of the newly constituted Board held recently, Dr. James Haig Ferguson, F.R.C.S.E., etc., was appointed Chairman, and Dr. A. Campbell Munro, D.Sc., etc., Deputy-Chairman. The following results of the examinations held simultaneously at Edinburgh, Glasgow, Dundee, and Aberdeen were reported: At Edinburgh there were forty-two candidates, of whom thirty-seven passed; at Glasgow there were fifty-three candidates, of whom forty-four passed; at Dundee there were fourteen candidates, of whom all passed; at Aberdeen there were seven candidates, of whom six passed.

